# Supplementary material for: Integrator dynamics in the cortico-basal ganglia loop for flexible motor timing
Source: Nature. 2025 Nov 19;649(8099):1244–53. doi: 10.1038/s41586-025-09778-2 (PMC12851927; doi:10.1038/s41586-025-09778-2)
Supplement: Supplementary file 1 — A supplementary discussion on modelling and tests distinguishing ‘pause’ versus ‘rewind’ effects, explanations of no-lick trials, alternative mechanisms for ramping activity, further characterization of the behaviour, striatum anatomy, PCA and mode comparisons, and summaries of optogenetic effects on behaviour and spiking activity. This file contains 8 Supplementary Figures. [file 41586_2025_9778_MOESM1_ESM.pdf]

nature portfolio

https://doi.org/10.1038/s41586-025-09778-2

Supplementary information

Integrator dynamics in the cortico-basal ganglia loop for flexible motor timing

In the format provided by the authors and unedited

Supplementary Discussion

Table of contents

I. Modeling and Testing Pause vs. Rewind Effects of Optogenetic Manipulations

1-1: Modeling the Effects of Optogenetic Manipulations on an Internal Timer -P. 2

1-2: Inhibiting ALM and D1-SPN at Different Time Points to Test Pause vs. Rewind -P. 3

1-3: Explanation of D1-SPN Inhibition-dependent Increase in No-lick Rate -P. 4

Supplementary Fig. 1: ALM recording during manipulation at different onsets

II. Evaluation of Alternative Mechanisms Underlying Ramping Activity - P.6

Supplementary Fig. 2: Schema of ramping activity in the initial-condition-driven regime

III. Additional Figures

Supplementary Fig. 3: Characterization of lick-time distribution in the lick-timing task -P. 8

Supplementary Fig. 4: Characterization of orofacial movements in the lick-timing task -P. 10

Supplementary Fig. 5: Striatal anatomy and recording -P. 12

Supplementary Fig. 6: Angle between PC and modes -P. 13

Supplementary Fig. 7: Summary of the behavioral effects observed across optogenetic manipulations -P. 14

Supplementary Fig. 8: Summary of optogenetic effect on spiking activity -P. 16

I. Modeling and Testing Pause vs. Rewind Effects of Optogenetic Manipulations

We reported that although the premotor cortex and striatum exhibit similar neural dynamics, their inhibition leads to distinct effects on timing behavior and neural dynamics, which we referred to as "pause" and "rewind." To rigorously distinguish these two effects in a hypothesis-driven manner, we conducted computational modeling followed by targeted experiments for validation.

1-1: Modeling the Effects of Optogenetic Manipulations on an Internal Timer

We modeled the 'timer' as a generic accumulator, whose dynamics can be perturbed in ways that mimic optogenetic manipulations - either by slowing or reversing accumulation (Fig. 6a). The 'timer' infers the passage of time by integrating a constant input or periodic event, as in an hourglass. This framework makes it explicit how distinct perturbation types (i.e. pause vs. rewind) reshape internal time tracking.

In this analogy, time is represented by the rising sand level in the bottom chamber, reflecting the accumulation (or temporal integration) of a constant sand flow, and when the sand reaches a threshold level, it triggers a lick. We varied inflow rates across trials so that the lick-time distribution under control conditions followed an inverse Gaussian distribution, consistent with the data. In addition to lick-time distribution, we analyzed lick hazard rate: the moment-by-moment likelihood of a lick occurring, given that it has not yet occurred. This measure reflects the instantaneous drive to lick, allowing us to analyze the temporal dynamics of lick probability.

Then, we simulated how two types of transient perturbations, **pause/slowdown** and **rewind**, influence behavior across trials. The models are circuit-agnostic, allowing for the interpretation of behavior without assuming a specific network implementation.

Pause/slowdown (Fig. 6b) corresponds to reducing the sand inflow by pinching the bottleneck of the hourglass. The extent of slowdown is scaled by a speed coefficient representing the strength of the optogenetic manipulation, where 1 indicates normal flow (no manipulation) and 0 represents a complete pause. Intermediate values indicate proportionally slower accumulation. Thus, here pause and slowdown lie on a continuum of varying manipulation strengths. During manipulations belonging to this category, the ramping increase in sand level stops (or slows down) and then resumes from a level close to (or above) the pre-perturbation level, depending on the speed coefficient. This causes a parallel shift in the lick-time distribution and lick hazard rate, equal to (if completely paused) or shorter than the manipulation duration. Importantly, the extent of this shift does not depend on when the manipulation starts, i.e., <u>state-independent</u>. In addition, the hazard rate may decrease during the manipulation, but recovers to the pre-perturbation level at the end of the manipulation, as the timer's internal state recovers close to its pre-perturbation state.

Rewind (Fig. 6c), in contrast, is modeled as flipping the hourglass (the rate of sand flow after the flip may differ from the original inflow rate, depending on the strength of the optogenetic manipulation). In this condition, the sand level in the original bottom chamber gradually decreases during the manipulation and then resumes from the reduced level once the manipulation ends (i.e., when the hourglass is flipped back). This causes a larger shift in both the lick-time distribution and hazard rate than the manipulation duration. Importantly, different from pause/slowdown, these shifts are <u>state-dependent</u>: the later the manipulation starts, the greater the shift. This state-dependency arises from a floor effect: once the timer rewinds to zero, it cannot rewind further (thus resulting in a functional 'reset' of the accumulator). This aligns with our recording showing that ramping activity does not fall below baseline (Fig. 6). In addition, unlike pause/slowdown, the hazard rate is 0 at the end of manipulation, as the timer has rewound and must begin tracking the passage of time before the lick again.

These distinct behavioral signatures — shift extent, hazard rate recovery, and state dependence — distinguish pause/slowdown from rewind. While the brain's internal time representation (e.g., ramping

activity) may reflect the modeled timer's dynamics, not all behavioral readouts are equally powerful to separate the effect of perturbations. Specifically, shift magnitude (used in the manuscript) is a quantitative measure that depends on the strength of the perturbation. In contrast, state dependence and hazard rate recovery represent qualitative signatures, offering a more robust means of distinguishing pause/slowdown from rewind.

1-2: Inhibiting ALM and D1-SPN at Different Time Points to Test Pause vs. Rewind Effects

Guided by the model's predictions described in **section 1**, we conducted experiments inhibiting ALM or D1-SPN at two time points after the cue (0.6 and 0.9 s after cue onset).

ALM silencing

Transient ALM silencing shifted the lick-time distribution and hazard rate (Fig. 6de) close to the duration of silencing (Fig. 6g). Importantly, the lick-time distribution and shift in hazard rate after silencing were indistinguishable between the two onset times (Fig. 6d-g), and the hazard rate recovered to the pre-silencing level by the end of the silencing period (Fig. 6h). Silicon probe recordings during these behavioral experiments confirmed effective ALM silencing, and ALM activity quickly returned to pre-perturbation level at the end of the manipulation, regardless of onset time (Supplementary Fig. 1ab). This pattern matches the timer's state in the pause/slowdown model, except for the collapse during silencing (this pattern is expected if ALM provides input to a downstream integrator, i.e., the striatum, rather than performing the temporal integration itself). Weak ALM silencing produced a smaller shift in lick time: 0.23 s (Extended Data Fig. 12ab), consistent with a milder slowdown. Together, the effect of ALM silencing is consistent with a pause/slowdown, with the extent of the slowdown depending on laser power (approaching a pause at the highest power we used). The pause/slowdown supports the idea that ALM provides input to the integrator, analogous to sand inflow in the hourglass.

D1-SPN inhibition

Transient D1-SPN inhibition shifted lick timing and hazard rate (Fig. 6i-k) longer than the duration of the manipulation (Fig. 6l). In contrast to ALM silencing, in this case, the shift was significantly larger when inhibition began later (0.9 s after the cue; Fig. 6l), and the lick-time distributions were distinct between the two onset times (Fig. 6i-k). During inhibition, the hazard rate dropped and remained at zero even at the end of the inhibition (Fig. 6m). Additionally, ALM ramping activity gradually decayed during D1-SPN inhibition and did not rapidly recover afterward, mirroring the internal time representation in the rewind model (Supplementary Fig. 1cd). Thus, the effect of D1-SPN inhibition is consistent with a rewind of the timer. This supports the idea that D1-SPN plays a key role in temporal integration, analogous to the bottom chamber in the hourglass.

To summarize, all behavioral measures and recording data consistently support the interpretation that ALM and striatal inhibition result in pause (slowdown when laser power is weak) and rewind of the timer, respectively.

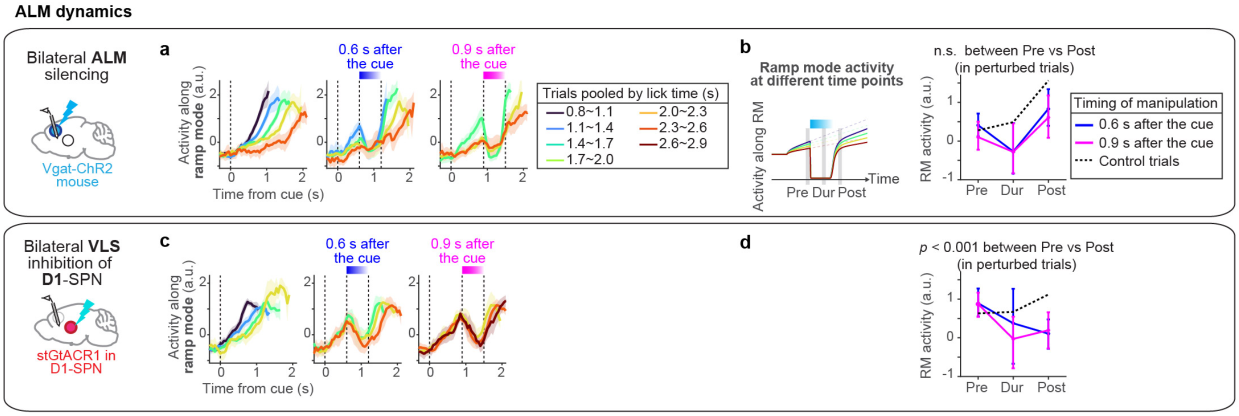

Supplementary Fig 1. ALM recording during manipulation at different onsets

a. *ALM dynamics during ALM silencing at different delay onsets. n = 15 sessions, 8 mice, same for b.*

b. *ALM population activity along the ramp mode before, during, and after the manipulation. ALM activity recovered to the pre-silencing level regardless of the silencing onsets. Data are presented as median values ± 95% CI, same for d. P-value, hierarchical bootstrap (null hypothesis: RM activity post-perturbation is bigger than or equal to pre-perturbation).*

c-d. *Same as* ***a-b*** *but for D1-SPN inhibition at different delay onsets. n = 7 sessions, 7 mice. In both manipulation onsets, ALM activity did not recover to the pre-silencing level.*

1-3: Explanation of D1-SPN Inhibition-dependent Increase in No-lick Rate

D1-SPN inhibition caused an increase in no-lick trials, in addition to the significant shift in lick timing. Here, we test the hypothesis that no-lick trials result from a rewinding of the internal timer, rather than representing an independent phenomenon.

Hypothesis and predictions: At the cue onset that initiates the internal 'timer', both ALM and the striatum exhibit dramatic changes in activity patterns. If timing dynamics rewind back to baseline, it may be difficult to restart the timer, as there is no second cue to re-initiate it. This could lead to a <u>stochastic failure to resume timing dynamics once activity returns to baseline</u>. If so, no-lick trials following D1-SPN inhibition should be those that are more likely to reach the baseline during inhibition, i.e., trials that start with lower pre-perturbation activity. Therefore, it should be possible to predict no-lick outcomes from neural activity preceding the perturbation (**Prediction I**). In addition, longer D1-SPN inhibition, which brings activity closer to baseline, should increase the rate of no-lick trials, in addition to producing a greater shift in lick timing (**Prediction II**).

To test **Prediction I**, we decoded the time to lick on individual trials using a k-nearest neighbors (kNN) decoder and compared lick and no-lick trials prior to inhibition onset (Extended Data Fig. 10ab). Consistent with our hypothesis, we can predict no-lick trials based on the activity preceding the perturbation: the decoded time to lick was significantly later in no-lick trials, consistent with these trials starting with lower pre-perturbation activity.

To test **Prediction II**, we varied the duration of inhibition (we reduced the laser power to 0.15 mW to avoid rebound effects associated with prolonged strong inhibition; Extended Data Fig. 10c-f). Consistent with our prediction, the no-lick rate increased as a function of inhibition duration, and was accompanied by a larger shift in lick timing (Extended Data Fig. 10c-e). Simultaneous ALM recordings revealed that D1-SPN inhibition caused a gradual decay of ramping activity during manipulation (Extended Data Fig. 10f). This decay continued with longer inhibition, leading to no-lick trials even when ramping activity was high prior to inhibition onset (Extended Data Fig. 10f-h), as the inhibition brought activity close to baseline (Extended Data Fig. 10i). This is consistent with our hypothesis that extended inhibition pushed activity closer to baseline, increasing the likelihood of a no-lick outcome.

As a control, we also varied the duration of ALM silencing (Extended Data Fig. 10j-l). While longer silencing led to larger shifts in lick timing (as expected for 'pause'), it did not increase the no-lick rate. Thus, not all manipulations that cause larger shifts in lick timing lead to an increase in the no-lick rate.

Altogether, the increase in no-lick trials is specific to D1-SPN inhibition and is likely due to its effect of driving the timing dynamics back to baseline. Thus, both extended the shift in lick timing and increased proportion of no-lick can be explained within the same framework of timing dynamics decaying back to baseline. We also note that if returning to baseline always led to a no-lick outcome, there would be no state dependency. However, if the no-lick outcome is stochastic or requires a prolonged stay at baseline, it can give rise to both state dependency and no-lick as implemented in Fig. 6c (Methods).

Summary

Together, these new results demonstrate that ALM and D1-SPN manipulations produce qualitatively different effects on the internal timer, as evidenced by both behavior and neural dynamics. In addition, our results confirm that the distinct effects of ALM and D1-SPN manipulations on lick timing and cortical dynamics are robust across different onset times and manipulation durations.

II: Evaluation of Alternative Mechanisms Underlying Ramping Activity

Here, we consider the question of whether there are alternative circuit-level mechanisms that could also explain our data.

In any recurrent network, activity evolves based on both initial conditions and external inputs. Depending on the network regime, which is shaped by both the strength and the structure of recurrent and external connections, one factor may dominate. In *input-driven* regimes, sustained external inputs primarily determine how activity evolves, as in integrator models. In contrast, in *initial-condition-driven* regimes, a brief input at the cue onset may set the initial state, but the subsequent dynamics unfold autonomously. Examples of this regime include ramping activity generated by slow drift toward a point attractor, or by runaway excitation (Supplementary Fig. 2a). In these relatively simple autonomous ramping models, ramping dynamics do not scale with time because trajectories are fixed once initiated. Ramping slopes remain the same across conditions with different action timing, except for the initial response to external input (Supplementary Fig. 2a), and therefore cannot account for the temporal scaling in dynamics observed during timing tasks (Fig. 3).

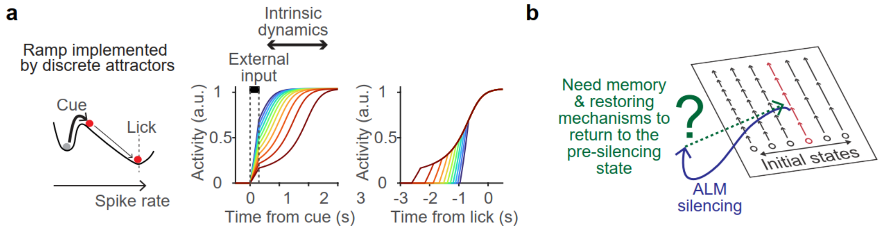

Supplementary Fig. 2. Schema of ramping activity in initial-condition-driven regime

a. *Ramping activity generated by discrete attractors. At cue onset, input drives the activity away from the baseline attractor, and the dynamics unfold toward the other attractor via a positive feedback loop with nonlinearity. The resulting ramping activity is identical across conditions, except for the initial externally driven increase, i.e., there is no temporal scaling.*

b. *To implement temporal scaling, multiple such attractors can be arranged in parallel in state space (black arrows), with the initial condition determining which trajectory unfolds (red). This can implement temporal scaling, yet, requires an additional mechanism to explain recovery to pre-perturbation level following silencing (green).*

To implement temporal scaling, initial-condition-driven models need to be extended by incorporating parallel trajectories in a higher-dimensional state space, where different initial conditions lead to trajectories that unfold at different speeds[4,79] (Supplementary Fig. 2b). However, to our knowledge, these extended models cannot intrinsically recover from transient silencing without an additional memory mechanism that stores and restores the internal state to its pre-perturbation level (as observed during ALM silencing). Such a memory mechanism would need to store a continuous state, which may be implemented by a mechanism analogous to an integrator. Thus, while these models can account for temporal scaling, they cannot explain recovery in timing dynamics after transient ALM silencing without additional complexity.

In contrast, input-driven integrator models naturally account for both temporal scaling and recovery. Varying the input strength modulates the ramping speed, thereby implementing temporal scaling. On-

manifold perturbations (aligned with the integration axis) that cancel out the input can pause the ramp, while off-manifold perturbations (orthogonal to the integrating axis) allow rapid recovery to the pre-perturbation state without requiring additional mechanisms. Thus, while it is possible that initial conditions also play a role in shaping neural dynamics, integrator-based architectures provide a more parsimonious explanation for both robustness to perturbation and the temporal scaling observed in our experiments.

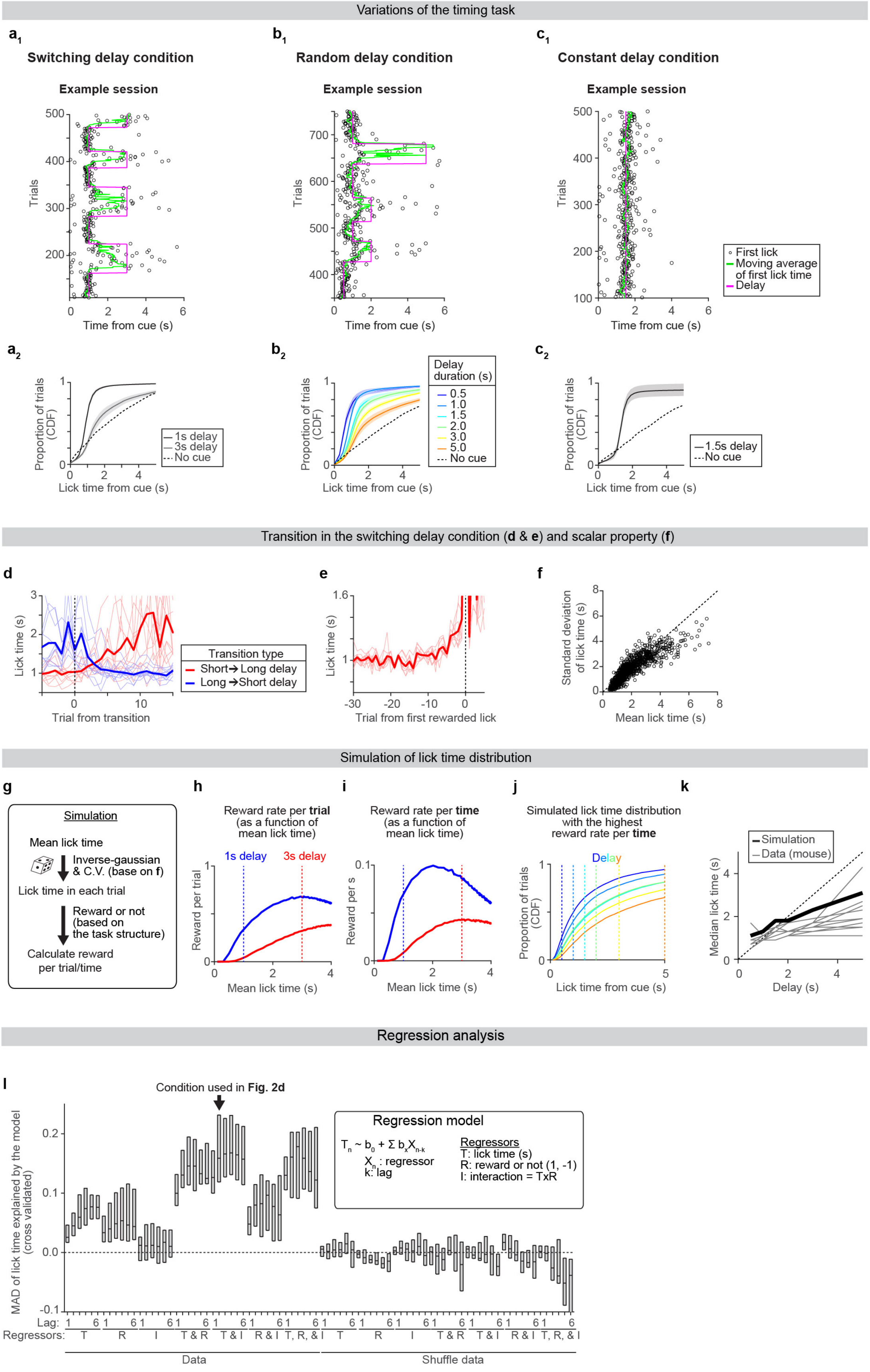

Variations of the timing task
a1
Switching delay condition
Example session
b1
Random delay condition
Example session
c1
Constant delay condition
Example session
Trials
Time from cue (s)
First lick
Moving average of first lick time
Delay
a2
b2
c2
Proportion of trials (CDF)
Lick time from cue (s)
1s delay
3s delay
No cue
Delay duration (s)
0.5
1.0
1.5
2.0
3.0
5.0
No cue
1.5s delay
No cue
Transition in the switching delay condition (d & e) and scalar property (f)
d
e
f
Lick time (s)
Trial from transition
Transition type
Short→Long delay
Long→Short delay
Trial from first rewarded lick
Standard deviation of lick time (s)
Mean lick time (s)
Simulation of lick time distribution
g
h
i
j
k
Simulation
Mean lick time
Inverse-gaussian & C.V. (base on f)
Lick time in each trial
Reward or not (based on the task structure)
Calculate reward per trial/time
Reward rate per trial (as a function of mean lick time)
1s delay
3s delay
Reward per trial
Mean lick time (s)
Reward rate per time (as a function of mean lick time)
Reward per s
Simulated lick time distribution with the highest reward rate per time
Delay
Proportion of trials (CDF)
Lick time from cue (s)
Simulation
Data (mouse)
Median lick time (s)
Delay (s)
Regression analysis
l
Condition used in Fig. 2d
MAD of lick time explained by the model (cross validated)
Regression model
T_n ~ b_0 + Σ b_k X_n-k
X_n : regressor
k: lag
Regressors
T: lick time (s)
R: reward or not (1, -1)
I: interaction = TxR
Lag:
Regressors:
T
R
I
T & R
T & I
R & I
T, R, & I
Data
Shuffle data

Supplementary Fig 3. Characterization of lick-time distribution in the lick-timing task

*We either changed the delay duration in blocks of trials (**a**, switching delay condition, when switching between two delays; **b**, random delay condition, when switching across multiple delays) or kept the delay identical across sessions/trials (**c**, constant delay condition). When delays switched, mice adjusted their lick times within 10 trials (**d, e**). The lick-time distribution exhibited scalar properties, similar to many other timing tasks across species (**f**). Mice often licked earlier than the delay duration, not maximizing reward per trial. However, this lick-time distribution (especially in the best-performing mice) is close to the simulated distribution that maximizes the reward amount per time, given the short inter-trial interval and the inverse normal distribution of lick times[80–84] (**g-k**, see below). 42 regression models were screened to identify the best model explaining the lick time (**l**).*

*In **g-k**, We estimated the optimal lick time in the timing task. Based on the mean and standard deviation (std) of the lick-time distribution in the data (std lick time = -0.2 + 1.1 × mean lick time; **f**), and assuming an inverse Gaussian distribution of lick time[80–84], we randomly sampled hypothetical lick times in 1000 trials for a given mean lick time. Then, following the task structure (identical to that described in **Methods**), we determined whether the agent would receive water or not for each trial and calculated the estimated reward amount per trial or time.*

- ***a.*** *lick-time distribution under switching delay condition. Example session (**a1**). Cumulative distribution of lick time in 1 s and 3 s delay blocks (**a2**). Duplicated from Figure 2bc for comparison. Shades, SEM (hierarchical bootstrap).*
- ***b.*** *Same as in **a** but for the random delay condition. n = 276 sessions, 17 mice.*
- ***c.*** *Same as in **a** but for the constant delay condition. n = 71 sessions, 13 mice.*
- ***d.*** *Change in lick time after transitioning between 1 and 3 s delay blocks. 0, last trial before delay transition. Thick lines, the mean across mice. Thin lines, individual mice (n =10 mice).*
- ***e.*** *Change in lick time before the first rewarded lick following transitions from a short to a long delay. Thick line, the mean across mice. Thin lines, individual mice (n = 10 mice).*
- ***f.*** *Relationship between the mean and the standard deviation of lick time. Circles, individual sessions (n = 153 sessions, 30 mice). The mean and standard deviation of lick time are correlated, consistent with scalar properties reported across species[85].*
- ***g.*** *Simulated lick-time distribution following an inverse-Gaussian distribution and coefficient of variation (CV) in **f**. Subsequently, based on the task structure, we calculated the reward rate (Methods).*
- ***h.*** *Simulated reward rate per **trial** as a function of mean lick time in 1 s delay block (blue) and 3 s delay block (red).*
- ***i.*** *Simulated reward rate per **time** as a function of mean lick time. Note that the peak reward rate is attained with a shorter mean lick time compared to that in **h**.*
- ***j.*** *Following the procedure described in **g** and **i**, we estimated the optimal mean lick time that yielded the highest reward rate per unit of time for each delay duration (delay duration is indicated by colored vertical dotted lines). We plotted the distribution of simulated lick times in these conditions. Note that in a large proportion of trials, the licks occurred before the end of the delay period, replicating what was observed in the data.*
- ***k.*** *Thick line, the median lick time of simulated optimal lick-time distribution in **j**. Thin lines, experimental data (individual mice). The optimal lick time appears to align with the upper limit of the experimental data.*
- ***l.*** *Median absolute deviation (MAD) of lick time explained by different trial-history regression models following cross-validation under the random delay condition (n = 276 sessions, 17 mice; results were consistent in the switching delay condition). Regressors and lags in each model are indicated at the bottom. Arrow, the condition that best explained the data (Fig. 2d). The central line in the box plot, median. Top and bottom edges, 75% and 25% points.*

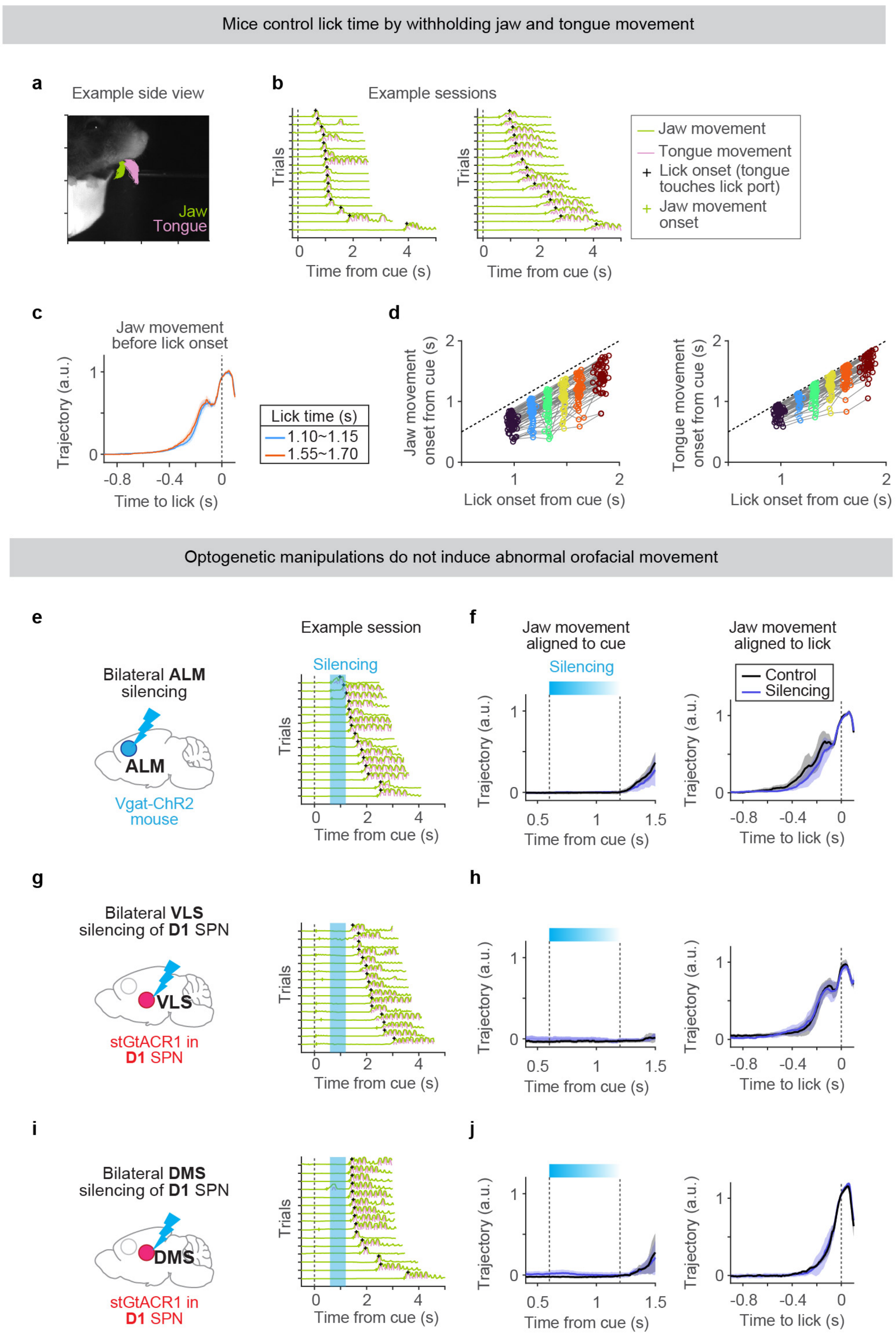

Mice control lick time by withholding jaw and tongue movement
a
Example side view
Jaw
Tongue
b
Example sessions
Trials
Time from cue (s)
Jaw movement
Tongue movement
Lick onset (tongue touches lick port)
Jaw movement onset
c
Jaw movement before lick onset
Trajectory (a.u.)
Time to lick (s)
Lick time (s)
1.10~1.15
1.55~1.70
d
Jaw movement onset from cue (s)
Lick onset from cue (s)
Tongue movement onset from cue (s)
Optogenetic manipulations do not induce abnormal orofacial movement
e
Example session
Bilateral ALM silencing
ALM
Vgat-ChR2 mouse
Silencing
f
Jaw movement aligned to cue
Jaw movement aligned to lick
Control
Silencing
g
Bilateral VLS silencing of D1 SPN
VLS
stGtACR1 in D1 SPN
h
i
Bilateral DMS silencing of D1 SPN
DMS
stGtACR1 in D1 SPN
j

Supplementary Fig 4. Characterization of orofacial movements in the lick-timing task

*Jaw and tongue movements were tracked using high-speed videography and DeepLabCut[86] (**a**). This revealed that mice adjusted lick times (detected by contact of the tongue with the lick port) by controlling the onset rather than the speed of tongue/jaw movements (**b-d**). Additionally, we did not detect any abnormal movements during/after ALM silencing (**e, f**), VLS inhibition (**g, h**), and DMS inhibition (**i, j**).*

*Methods: High-speed (300 Hz) videography of orofacial movement (side view) was acquired using a CMOS camera (Chameleon3 CM3-U3-13Y3M-CS, FLIR) with IR illumination (940nm LED). We used DeepLabCut[86] to track the movement of the tongue and jaw. Movements along the dorsoventral direction were analyzed and plotted. Trajectories were normalized by subtracting the mean position before the cue from each trajectory and then dividing by the minimum value within a session (thus, the downward movement of the tongue and jaw looks upward in the plot). The onset of jaw movement in each trial is the first time point after the cue when the normalized movement trajectory exceeds 10% of the max value. The onset of tongue movement is when DeepLabCut first detects the tongue after the cue. In two out of 34 mice, mice moved jaws within 200 ms after the cue in some trials. These trials were excluded from the analysis for the average jaw and tongue onset analyses (**d**), as these rare early subthreshold movements are likely startled responses to the cue.*

a. *An example side-view clip of a mouse. Movements of the jaw (green) and tongue (purple) were tracked. Trajectories of individual trials are overlaid.*

b. *Vertical jaw (green) and tongue (purple) movements in two example sessions. The left session is from the animal shown in **a**. 15 randomly selected trials sorted by lick time are shown.*

c. *Average trajectories of vertical jaw movement aligned to the lick onset. The kinematics of jaw movement remain consistent regardless of the lick timing (indicated in different colors). Lines, grand median. Shading, SEM (bootstrap). n = 58 sessions, 34 mice.*

d. *Relationship between lick onset (the timing when the tongue contacted the lick port) and jaw movement onset (left) or tongue movement onset (right). Trials were grouped into six ranges. The onset of movement was tightly correlated with lick onset. Circles, individual mice. Dotted line, the unity line. n = 58 sessions, 34 mice. **c-d** conclude that mice did not change their kinematics but instead the onset of movement when they lick at different timings. n = 58 sessions, 34 mice.*

e. *Tongue and jaw movement trajectories in an example session with ALM silencing. Same format as in **b**. Cyan bar, silencing.*

f. *Jaw movement aligned to the cue (left) or the lick onset (right). Trials with lick after the silencing were analyzed. Lines, grand median. Shading, SEM (bootstrap). n = 26 sessions, 9 mice. No abnormal movement was detected during silencing, and the animals followed normal kinematics to lick even in the silencing trials.*

g-h. *Same as in **e-f**, but with D1 VLS silencing. n = 6 sessions, 6 mice.*

i-j. *Same as in **e-f**, but with D1 DMS silencing. n = 6 sessions, 6 mice.*

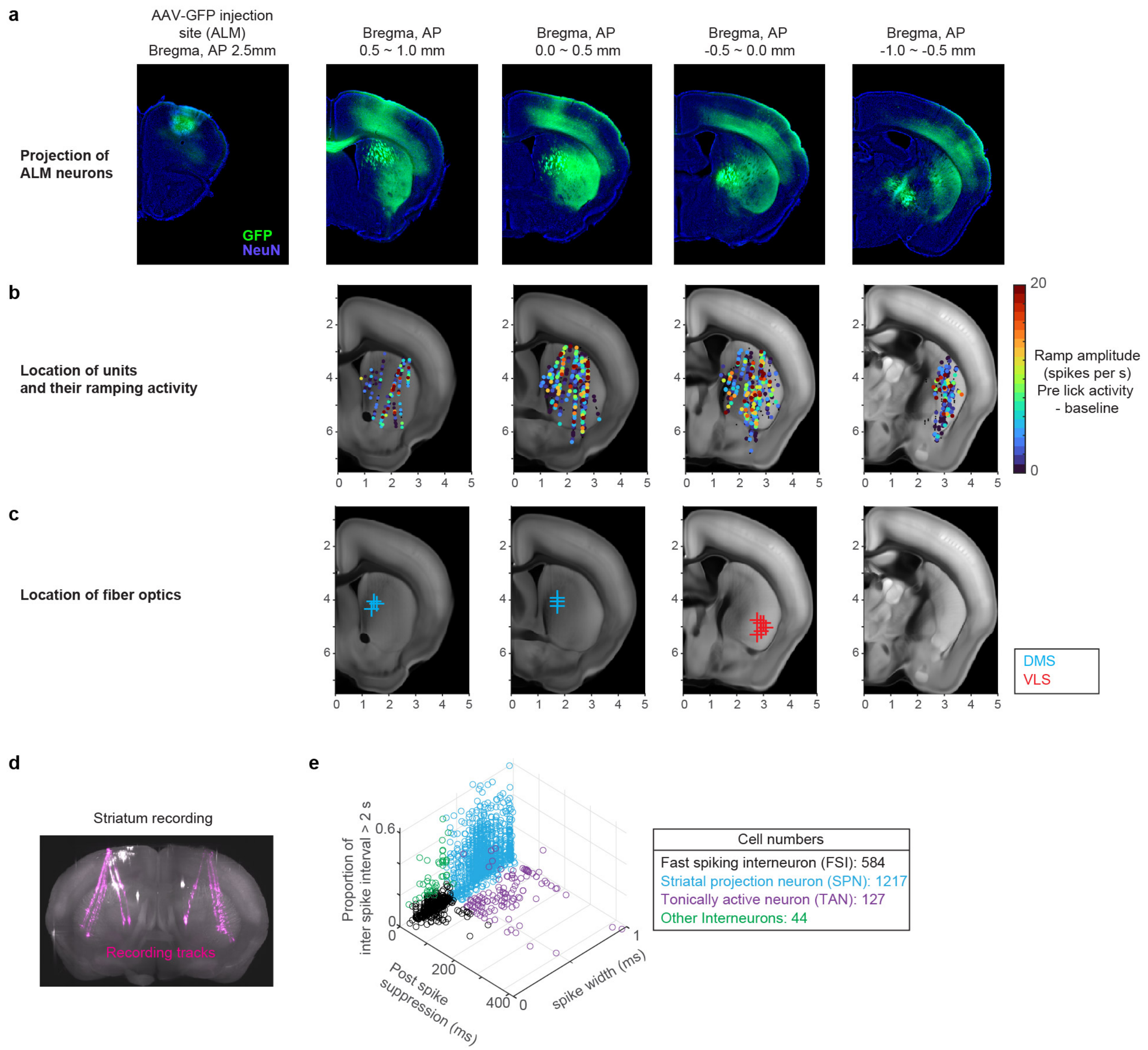

Supplementary Fig 5. Striatal anatomy and recording

*ALM projects to a large portion of the striatum (**a**), and we observed task-modulated activity across these sectors (**b**). See **d** for example recording tracks and **e** for the classification of cell types based on spike features.*

a. *ALM neurons project across sectors in the striatum. AAV-GFP was injected into ALM. This based on a brain imaged in Guo et al, 2017[87].*

b. *The spatial distribution of recorded striatal neurons in the Allen CCF. Colors, the extent of increase in spiking activity before the lick compared to the baseline. Black dots, neurons that do not ramp up.*

c. *Locations of the tips of the tapered fiber optics implanted for bilateral striatal silencing (only showing one hemisphere as the two fibers were implanted symmetrically). Related to Fig. 5.*

d. *An example brain image of recording tracks acquired by a light sheet microscopy (Methods). Striatal recording tracks are labeled with CM-DiI (magenta). Coronal view, maximal intensity projection of 415 µm optical section.*

e. *Striatal cell types were classified based on three spike features[44] (Methods).*

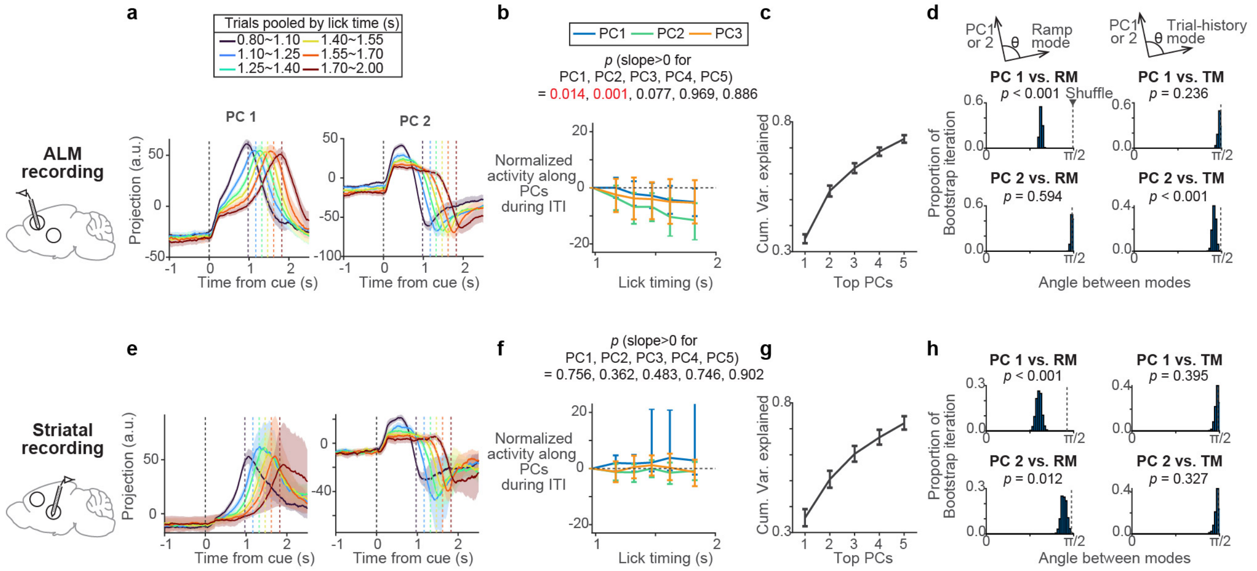

Supplementary Fig 6. Angle between PC and modes

*The top 2 PCs in ALM closely resembled and aligned with the ramp mode (PC1) and trial-history mode (PC2), respectively (**a**, **b**, and **d**), indicating that ramp and trial-history modes are not arbitrary patterns extracted by targeted dimensionality reduction, but rather reflect major activity patterns in ALM. In contrast, PC2 in the striatum lacked trial-history mode-like activity, consistent with a weak trial-history encoding (**e** and **f**). Note that in high-dimensional spaces, small perturbations can induce large angular deviations between vectors, typically driving them toward orthogonality. Thus, the key comparison is between shuffled and actual data (**d** and **h**).*

Method: we performed PCA on ALM and striatal activity from trials in which the lick occurred between 0.8 and 1.1 s. We constructed an $n \times T$ matrix, where n is the number of recorded neurons and T is the time points between -1 and 1.05 s from cue. Each row (i.e., each neuron's activity across time) was z-scored, and PCA was then applied to this matrix. The resulting principal components, derived from this specific trial type, were used to project activity from trials with different lick times.

- ***a.*** *Projection of ALM activity to PC1 (left) and PC2 (right). Shade, 95% CI. n = 3261 neurons, 45 mice, same for **b** and **c**.*
- ***b.*** *Relationship between actual lick time and amplitude of activity along each PC during ITI (Amplitude is normalized to the activity in trials with the shortest lick times). Data are presented as mean ± 95% CI. P-values, bootstrap test of the null hypothesis that the linear regression slope between these two is larger than 0. Notably, PC2 shows a significantly negative slope, similar to the trial-history mode.*
- ***c.*** *Cumulative variance explained by PCs. Data are presented as mean ± 95% CI*
- ***d.*** *Angle between each PC and ramp mode or trial-history mode (histogram of 1000 bootstrap iterations is shown). Dotted lines, angles of shuffled modes. P-values, bootstrap test of the null hypothesis that the observed angle is equal to the shuffled angle. PC1 and PC2 are significantly non-orthogonal with ramp mode and trial-history mode, respectively.*
- ***e-h.*** *Same as **a-d** but for striatal activity (n = 1073 neurons, 16 mice). PC1 and PC2 are non-orthogonal with the ramp mode, but none of the top principal components aligned with the trial-history mode (we tested the top 5 PCs; only the top 2 are shown here).*

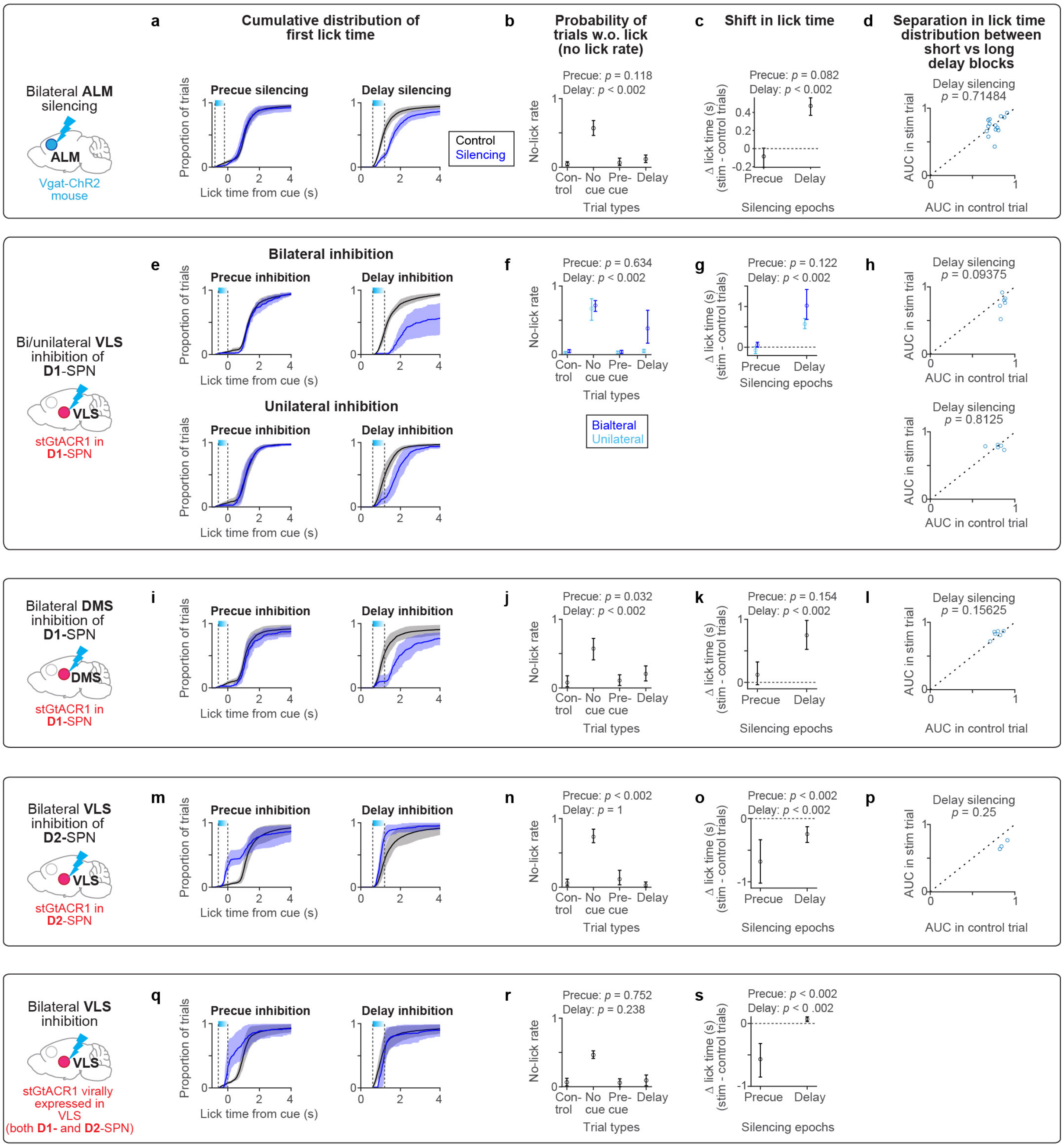

Supplementary Fig 7. Summary of the behavioral effects observed across optogenetic manipulations

a. Cumulative lick-time distribution during precue or delay ALM silencing, duplicated from Figure 4a for comparison. n = 14 mice, same for b-d

b. No-lick rate in control, no-cue, precue silencing, and delay silencing trials (Methods). No-cue trial refers to randomly interleaved trials without a cue, serving to monitor the spontaneous lick rate not triggered by a cue. This represents the upper bound of the no-lick rate. *P*-value, hierarchical bootstrap with a null hypothesis that no-lick rate in control trials is the same as in silencing trials. Data are presented as median values ± 95% CI, applied to all the panels with error bars in this figure.

c. Shift in median lick time caused by precue or delay silencing. Duplicated from Figure 4b for comparison. *P*-value, hierarchical bootstrap with a null hypothesis that there is no change in lick time.

d. ALM silencing does not affect the separation in lick-time distribution between delay blocks (short vs. long delay blocks in the switching delay condition). To analyze whether optogenetic manipulation affects the separation of lick-time distributions between different delay blocks in the switching delay condition (short vs. long delay blocks) we performed a receiver operating characteristic (ROC) analysis. First, we conducted ROC analysis to distinguish lick-time distributions between the two delay blocks (for control and photostimulation trials separately). We then quantified the area under the curve (AUC) to measure the separation in lick-time distributions between delay blcoks, and compared these values between control and photostimulation trials. Circles, individual animals. *P* value, two-sided signed-rank test. The absence of change in the separation of lick-time distributions suggests that ALM silencing does not erase the information of intended lick time, consistent with the recovery of dynamics after the silencing (Fig. 4).

e-h. Same as in **a-d** but for D1-SPN inhibition in VLS. Top, bilateral D1-SPN inhibition in VLS (n = 6 mice). Duplicated from Fig. 5 for comparison. Bottom, unilateral inhibition (n = 5 mice).

i-l. Same as in **a-d** for D1-SPN inhibition in DMS. n = 6 mice.

m-p. Same as in **a-d** for D2 SPN inhibition in VLS. n = 3 mice.

q-s. Same as in **a-c** for cell-type-nonspecific striatal silencing in VLS. n = 2 mice.

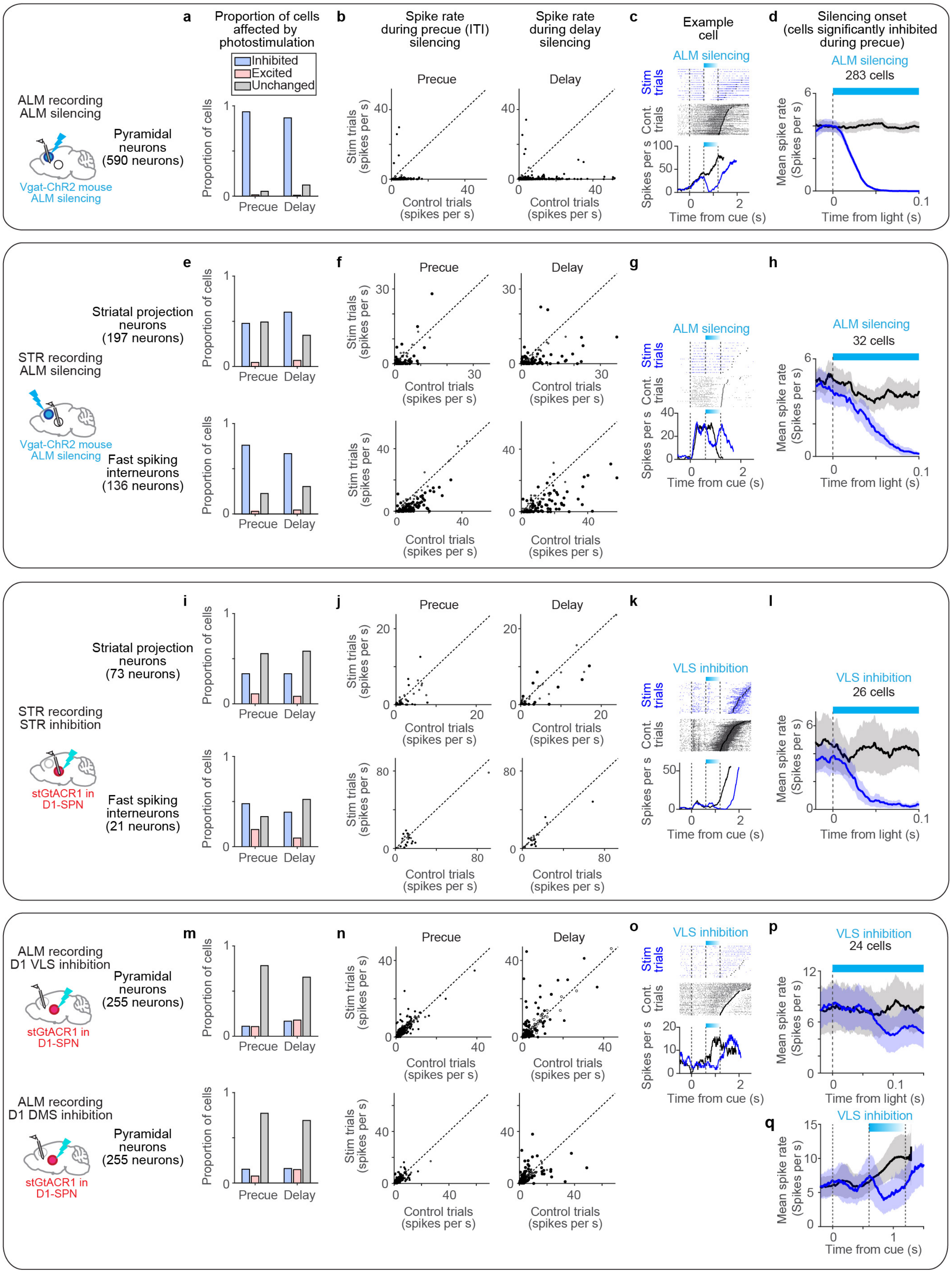

Supplementary Fig 8. Summary of optogenetic effect on spiking activity

*We characterized the effects of different optogenetic manipulations on spiking activity, including the proportion of inhibited/excited/unaffected cells (**a, e, i,** and **m**), changes in spike rate (**b, f, j,** and **n**), and the onset of the optogenetic effect in significantly inhibited neurons (analyzed during the precue epoch to avoid confounds caused by behavior-related activity; **d, h, l,** and **p**).*

a. *The proportion of ALM pyramidal neurons affected by bilateral ALM silencing was assessed. Neurons were categorized as inhibited or excited ($p < 0.05$) or unchanged ($p >= 0.05$) using a two-sided rank-sum test. Cells with a mean spike rate higher than 1 Hz in control trials during the photostimulation window were considered. Duplicated from Fig. 4c for comparison. Units from the first two manipulation sessions were included.*

b. *Spike rate of ALM neurons during precue (left) or delay silencing (right) compared to control unperturbed trials. Circles, cells. Filled circles, significantly affected cells ($p < 0.05$, two-sided rank-sum test). The spike rate was calculated between 50 - 250 ms from the photostimulation onset.*

c. *An example ALM neuron with a reduced spike rate during delay ALM silencing. Top, spike raster. Blue, silencing trials. Black, control trials. Bottom, PSTH up to the median lick time for each trial type. The spike rate was smoothed using a 200 ms boxcar causal filter.*

d. *The onset of the optogenetic effect in ALM neurons with a significant reduction in spike rate during ALM silencing ($p < 0.05$, two-sided rank-sum test). We analyzed silencing during the precue epoch to avoid confounds caused by behavior-related activity. Neurons with more than 10 trials and a spike rate higher than 1 Hz in the control condition were considered. The spike rate was smoothed using a 30 ms boxcar causal filter and aligned to the photostimulation onset. Blue, silencing trials. Black, control trials. Lines, grand mean. Shading, SEM (hierarchical bootstrap).*

e-h. *Same as in **a-d** but for striatal recording during ALM silencing. Top, striatal projection neurons. Bottom, striatal fast-spiking interneurons. Units from the first two manipulation sessions were included.*

i-l. *Same as in **a-d** but for striatal recording during D1-SPN unilateral inhibition. Top, striatal projection neurons. Bottom, striatal fast-spiking interneurons. Data from the first session was included for **i-k**. However, for **l**, data was pooled across 3 sessions, as the onset of silencing did not appear to change across days, despite significant behavioral changes likely caused by rebound or other activity changes after the silencing.*

m-p. *Same as in **a-d** but for ALM recording during D1-SPN inhibition in VLS (top), or in DMS (bottom). Units from the first manipulation session were included.*

q. *Same as in **p** but for D1-SPN inhibition during the delay. On average, ALM neurons inhibited by D1-SPN show ramping-up activity in the control condition, suggesting that D1-SPN specifically drives the ramping activity pattern in ALM.*

SUPPLEMENTARY REFERENCES

79. Remington, E. D., Narain, D., Hosseini, E. A. & Jazayeri, M. Flexible Sensorimotor Computations through Rapid Reconfiguration of Cortical Dynamics. *Neuron* **98**, 1005-1019.e5 (2018).
80. Kheifets, A. & Gallistel, C. R. Mice take calculated risks. *Proc. Natl. Acad. Sci.* **109**, 8776–8779 (2012).
81. Freestone, D. M., Balcı, F., Simen, P. & Church, R. M. Optimal response rates in humans and rats. *J. Exp. Psychol. Anim. Learn. Cogn.* **41**, 39–51 (2015).
82. Çavdaroğlu, B., Zeki, M. & Balcı, F. Time-based reward maximization. *Philos. Trans. R. Soc. B Biol. Sci.* **369**, 20120461 (2014).
83. Balcı, F. *et al.* Optimal Temporal Risk Assessment. *Front. Integr. Neurosci.* **5**, (2011).
84. Mitchell, S. H. Assessing delay discounting in mice. *Curr. Protoc. Neurosci. Editor. Board Jacqueline N Crawley Al* **66**, 8.30.1-8.30.12 (2014).
85. Gibbon, J. Scalar expectancy theory and Weber's law in animal timing. *Psychol. Rev.* **84**, 279–325 (1977).
86. Mathis, A. *et al.* DeepLabCut: markerless pose estimation of user-defined body parts with deep learning. *Nat. Neurosci.* **21**, 1281–1289 (2018).
87. Guo, Z. V. *et al.* Maintenance of persistent activity in a frontal thalamocortical loop. *Nature* **545**, 181–186 (2017).
