# Supplementary material for: Integrator dynamics in the cortico-basal ganglia loop for flexible motor timing
Source: Nature. 2025 Nov 19;649(8099):1244–53. doi: 10.1038/s41586-025-09778-2 (PMC12851927; doi:10.1038/s41586-025-09778-2)
Supplement: Supplementary file 2 — Reporting Summary [file 41586_2025_9778_MOESM2_ESM.pdf]

nature portfolio

Corresponding author(s): Hidehiko Inagaki

Last updated by author(s): Oct 3, 2025

Reporting Summary

Nature Portfolio wishes to improve the reproducibility of the work that we publish. This form provides structure for consistency and transparency in reporting. For further information on Nature Portfolio policies, see our Editorial Policies and the Editorial Policy Checklist.

Statistics

For all statistical analyses, confirm that the following items are present in the figure legend, table legend, main text, or Methods section.

n/a	Confirmed	
☐	☒	The exact sample size (n) for each experimental group/condition, given as a discrete number and unit of measurement
☐	☒	A statement on whether measurements were taken from distinct samples or whether the same sample was measured repeatedly
☐	☒	The statistical test(s) used AND whether they are one- or two-sided<br>*Only common tests should be described solely by name; describe more complex techniques in the Methods section.*
☐	☒	A description of all covariates tested
☐	☒	A description of any assumptions or corrections, such as tests of normality and adjustment for multiple comparisons
☐	☒	A full description of the statistical parameters including central tendency (e.g. means) or other basic estimates (e.g. regression coefficient) AND variation (e.g. standard deviation) or associated estimates of uncertainty (e.g. confidence intervals)
☐	☒	For null hypothesis testing, the test statistic (e.g. F, t, r) with confidence intervals, effect sizes, degrees of freedom and P value noted<br>*Give P values as exact values whenever suitable.*
☒	☐	For Bayesian analysis, information on the choice of priors and Markov chain Monte Carlo settings
☐	☒	For hierarchical and complex designs, identification of the appropriate level for tests and full reporting of outcomes
☐	☒	Estimates of effect sizes (e.g. Cohen's d, Pearson's r), indicating how they were calculated

Our web collection on statistics for biologists contains articles on many of the points above.

Software and code

Policy information about availability of computer code

Data collection	spikeGLX, BPod
Data analysis	Matlab R2020b, R2022b, Python 3, JRClust, DeepLabCut2.3.5

For manuscripts utilizing custom algorithms or software that are central to the research but not yet described in published literature, software must be made available to editors and reviewers. We strongly encourage code deposition in a community repository (e.g. GitHub). See the Nature Portfolio guidelines for submitting code & software for further information.

Data

Policy information about availability of data

All manuscripts must include a data availability statement. This statement should provide the following information, where applicable:

- Accession codes, unique identifiers, or web links for publicly available datasets
- A description of any restrictions on data availability
- For clinical datasets or third party data, please ensure that the statement adheres to our policy

The recording data in NWB format is available at the DANDI archive (ID 001610). Codes is available at https://github.com/inagaki-lab/Yang_et_al_2024

Research involving human participants, their data, or biological material

Policy information about studies with human participants or human data. See also policy information about sex, gender (identity/presentation), and sexual orientation and race, ethnicity and racism.

Reporting on sex and gender	N/A
Reporting on race, ethnicity, or other socially relevant groupings	N/A
Population characteristics	N/A
Recruitment	N/A
Ethics oversight	N/A

Note that full information on the approval of the study protocol must also be provided in the manuscript.

Field-specific reporting

Please select the one below that is the best fit for your research. If you are not sure, read the appropriate sections before making your selection.

☒ Life sciences ☐ Behavioural & social sciences ☐ Ecological, evolutionary & environmental sciences

For a reference copy of the document with all sections, see nature.com/documents/nr-reporting-summary-flat.pdf

Life sciences study design

All studies must disclose on these points even when the disclosure is negative.

Sample size	The sample sizes are matched to the standadrd in the field (~6 mice per condition; e.g., Li et al, Nature, 2015)
Data exclusions	Quality metrics were used to determine single units to be analyzed. See Methods for details.
Replication	All experiments were performed extensively, as reported in the manuscript (e.g., ALM silencing and D1 SPN silencing during delay were tested for 3 times , n> 4 mice each, independently) and hierarchical bootstrap was used to rigorously test for reproducibility among animals.
Randomization	All trial types were randomly determined by the program
Blinding	For all experiments (behavior and recording experiments) were fully controlled by computer programs and experimenters did not have control to bias results. Experimenters were blind to experiment during the spike sorting.

Reporting for specific materials, systems and methods

We require information from authors about some types of materials, experimental systems and methods used in many studies. Here, indicate whether each material, system or method listed is relevant to your study. If you are not sure if a list item applies to your research, read the appropriate section before selecting a response.

Materials & experimental systems

n/a	Involved in the study	
☒	☐	Antibodies
☒	☐	Eukaryotic cell lines
☒	☐	Palaeontology and archaeology
☐	☒	Animals and other organisms
☒	☐	Clinical data
☒	☐	Dual use research of concern
☒	☐	Plants

Methods

n/a	Involved in the study	
☒	☐	ChIP-seq
☒	☐	Flow cytometry
☒	☐	MRI-based neuroimaging

Animals and other research organisms

Policy information about studies involving animals; ARRIVE guidelines recommended for reporting animal research, and Sex and Gender in Research

Laboratory animals	We used five mouse lines: C57Bl/6J (JAX# 000664), VGAT-ChR2-EYFP87 (JAX #14548), Drd1-cre FK15074, Adora2-cre KG12674, R26-LNL-GtACR1-Fred-Kv2.188 (JAX #33089). See Extended Data Table 1 for mice used in each experiment. For all experiments, age > P60.
Wild animals	No wild animals were used in the study.
Reporting on sex	This study is based on both adult male and female mice (age > P60).
Field-collected samples	No field collected samples were used in the study.
Ethics oversight	All procedures were in accordance with protocols approved by the MPFI IACUC committee. Mice were housed in a 12:12 reverse light: dark cycle and behaviorally tested during the dark phase. Temp 74F. Humidity 35-60%

Note that full information on the approval of the study protocol must also be provided in the manuscript.

Plants

Seed stocks	N/A
Novel plant genotypes	N/A
Authentication	N/A
