# Supplementary material for: Integrator dynamics in the cortico-basal ganglia loop for flexible motor timing
Source: Nature. 2025 Nov 19;649(8099):1244–53. doi: 10.1038/s41586-025-09778-2 (PMC12851927; doi:10.1038/s41586-025-09778-2)
Supplement: Supplementary file 3 — Sample sizes (n) used across experiments. [file 41586_2025_9778_MOESM3_ESM.pdf]

Supplementary Table 1: sample sizes (n) in the paper

Experiment	Mice	Sessions	Total neurons	Putative pyramidal neurons / Striatal projection neurons	Corresponding figures
ALM recording under switching delay					
Cue intensity	5 (C57Bl/6J)	15	788	696	EDF.5g-j
ALM silencing	14 (VGAT-ChR2-EYFP)	28	711	590	Fig. 4c and f-i, EDF. 6a, and EDF. 7a-e, EDF. 8a-d, EDF. 11a-c, edf. 12c-f
ALM weak silencing	12 (VGAT-ChR2-EYFP)	23	485	425	EDF. 12c-f
VLS D1 inhibition	6 (Drd1-cre FK150 x R26-LNL-GtACR1-Fred-Kv2.1)	6	283	255	Fig. 5e, and l-o, EDF. 6e, EDF. 7k-o, EDF. 8m-p, EDF. 11d-f, and EDF. 12g-i
VLS DI inhibition at different durations	4 (Drd1-cre FK150 x R26-LNL-GtACR1-Fred-Kv2.1)	4	118	103	EDF.10f-i
VLS D1 unilateral inhibition	4 (Drd1-cre FK150 x R26-LNL-GtACR1-Fred-Kv2.1)	4	320	282	EDF. 12g-i
DMS D1 inhibition	6 (Drd1-cre FK150 x R26-LNL-GtACR1-Fred-Kv2.1)	6	287	255	Supplementary Fig. mn, bottom
VLS DI inhibition at different delay onsets	7 (Drd1-cre FK150 x R26-LNL-GtACR1-Fred-Kv2.1)	7	132	106	Supplementary Fig. 1cd
ALM silencing at different delay onsets	8 (VGAT-ChR2-EYFP)	15	174	158	Supplementary Fig. 1ab
Other recordings	8 (2 C57Bl/6J; 6 Adora2-cre KG126 x R26-LNL-GtACR1-Fred-Kv2.1) + all the mice above	108	3267	2892	N/A
The sum of all experiments	74	216	6565	5762	Fig. 3b-e, and l, EDF. 2b-d, ef, k-m, EDF. 3, EDF. 4, and EDF. 5a-d

Striatum recording under switching delay					
ALM silencing	7 (VGAT-ChR2-EYFP)	13	372	197	Fig. 4c, j-m, EDF. 7f-j, EDF. 8e-h, and EDF. 12g-i
VLS D1 unilateral silencing	5 (Drd1-cre FK150 x R26-LNL-GtACR1-Fred-Kv2.1)	10	103	73	Fig. 5e,h-k, EDF. 8i-l, and EDF. 12g-i
Other recordings	4 (VGAT-ChR2-EYFP) + all mice above	74	1497	947	N/A
The sum of all experiments	16	97	1972	1217	Fig. 3g-j, EDF. 2l and m, EDF. 3f-j and l, and EDF. 5bd,
ALM recording under constant delay					
Cue intensity	5 (C57Bl/6J)	15	977	855	EDF. 5l-o
Other recordings	13 (7 C57Bl/6J; 6 VGAT-ChR2-EYFP)	48	1632	1425	N/A
The sum of all experiments	18 (7 C57Bl/6J; 6 VGAT-ChR2-EYFP)	63	2609	2280	EDF. 2l, and EDF. 5cd

“Other recordings” include the unused sessions from the rows above. This is because we limited the analysis of perturbation experiments to the first 1 or 2 days of experiments for each animal, but the control trials in all sessions were used for the figures corresponding to the row “The sum of all experiments”.

Neurons used for analysis in the rows of “The sum of all experiments” were based on whether the neuron has more than 10 trials for all 6 lick time ranges: 0.80 - 1.10, 1.10 - 1.25, 1.25 - 1.40, 1.40 - 1.55, 1.55 - 1.70, 1.70 - 2.00 sec.
